# Supplementary material for: LC–MS Lipidomics: Exploiting a Simple High-Throughput Method for the Comprehensive Extraction of Lipids in a Ruminant Fat Dose-Response Study
Source: Metabolites. 2020 Jul 17;10(7):296. doi: 10.3390/metabo10070296 (PMC7407148; doi:10.3390/metabo10070296)
Supplement: Supplementary file 1 [file metabolites-10-00296-s001.pdf]

metabolites

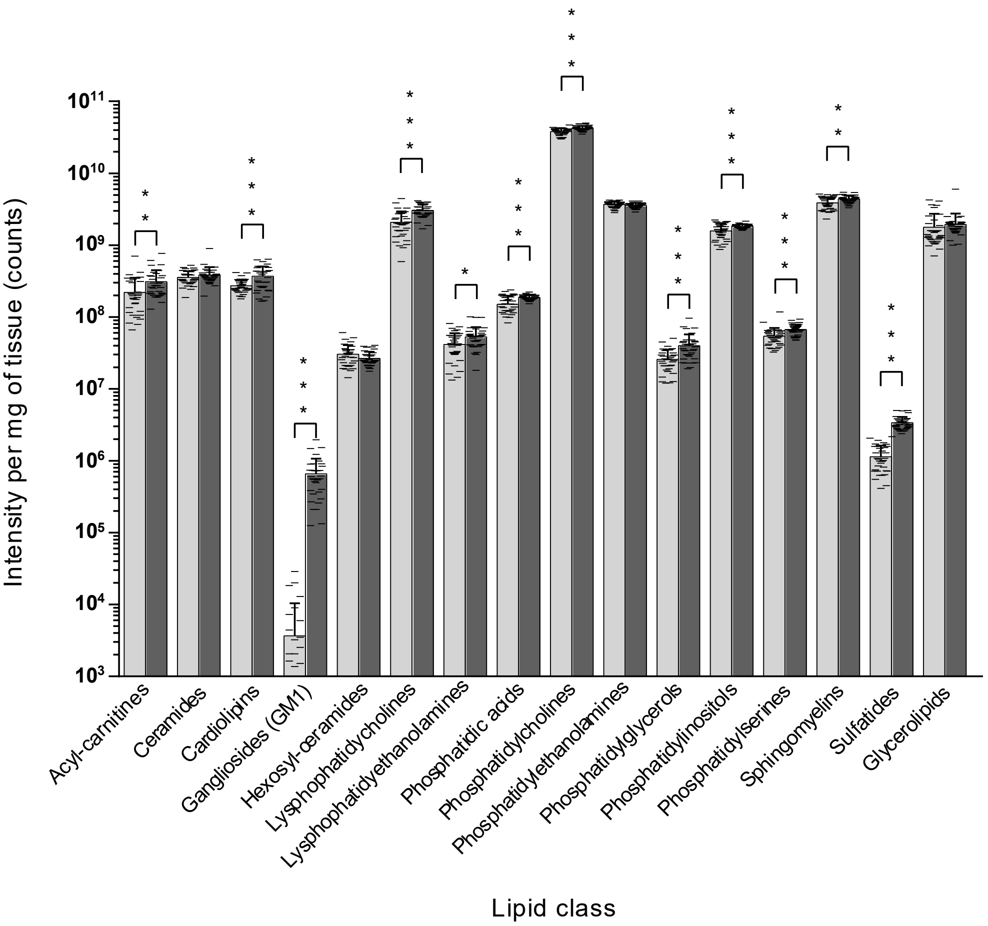

Figure S1. This figure shows the comparison between the two lipid extraction techniques regarding their extraction efficiency on each lipid class detected in the rat liver samples by each sample extraction method (Folch liquid-liquid extraction with a compositions of chloroform: methanol: water, ~7: 3: 4 , and Protein precipitation liquid extraction with a composition of chloroform: methanol: acetone, ~7: 3: 4). n = 34 rat liver samples per extraction method. The intensity of the lipids were measured by liquid chromatography with mass spectrometry and were not normalised to any internal standard. The significance of the difference between the two extraction protocols are shown by the p-value star system; where $p \leq 0.05$ was considered statistically significant ($p<0.05$ = *, $p<0.01$ = **, $p<0.001$ = ***). Error bars represent ± standard deviation.

Table S1. This table shows the comparison between the two lipid extraction techniques regarding their extraction efficiency on the total intensity of each lipid class detected in the rat liver samples by each sample extraction method (Folch-LLE: Folch liquid-liquid extraction with a compositions of chloroform: methanol: water, ~7: 3: 4, and PPLE: protein precipitation liquid extraction with a composition of chloroform: methanol: acetone, ~7: 3: 4). n = 34 rat liver samples per extraction method. The percentage increase the PPLE method is over the Folch-LLE method is shown (% diff.) along with the p value resulting from a t-test (p<0.05 designates statistical significance are in bold & shaded). The total number of lipid species detected and pass the quality control process are also shown.

	**Folch-LLE total lipid intensity**	**PPLE total lipid intensity**	**% diff.**	**P value**
**Acyl-carnitines**	**2.20E+08**	**3.11E+08**	**41%**	**0.00689**
Ceramides	3.92E+08	4.25E+08	8%	0.209
**Cardiolipins**	**2.72E+08**	**3.68E+08**	**35%**	**0.000347**
**Gangliosides (GM1)**	**3.67E+03**	**6.59E+05**	**18,000%**	**1.61E-13**
Hesosyl-ceramides	3.03E+07	2.66E+07	-12%	0.0824
**Lysophosphatidylcholines**	**2.07E+09**	**3.04E+09**	**47%**	**1.57E-06**
**Lysophosphatidylethanolamines**	**4.16E+07**	**5.32E+07**	**28%**	**0.0117**
**Phosphatidic acids**	**1.50E+08**	**1.86E+08**	**24%**	**8.26E-05**
**Phosphatidylcholines**	**3.80E+10**	**4.23E+10**	**11%**	**1.78E-05**
Phosphatidylethanolamines	3.68E+09	3.59E+09	-3%	0.235
**Phosphatidylglycerols**	**2.58E+07**	**3.99E+07**	**55%**	**7.05E-05**
**Phosphatidylinositols**	**1.58E+09**	**1.83E+09**	**16%**	**0.000833**
**Phosphatidylserines**	**5.40E+07**	**6.71E+07**	**24%**	**0.000315**
**Sphingomyelins**	**3.87E+09**	**4.39E+09**	**13%**	**0.00157**
**Sulfatides**	**1.14E+06**	**3.38E+06**	**197%**	**2.71E-23**
Glycerolipids	1.77E+09	1.94E+09	9%	0.441
Total No. of lipid species	455	472		

Table S2. This table shows the lipids quantified in this LC-MS method, along with the ionisation mode (either positive; +ve, or negative; -ve), the detected ion (m/z), the expected retention time (minutes) and the internal standard used for normalisation and quantification.. Lipid are shown in their shorthand notations with the number of carbons and unsaturated bonds in the fatty acid moiety separated by a colon; acyl-carnitines (Carn), ceramides (Cer), cardiolipins (CL), diacylglycerols (DG), gangliosides (GM1), hexosylceramides (Hex-Cer), lyso-phosphatidylcholines (LPC), lyso-phosphatidyethanolamines (LPE), lyso-phosphatidylinositols (LPI), lyso-cardiolipins (Lyso_CL), phosphatidic acids (PA), phosphatidylcholines (PC), phosphatidylethanolamines (PE), phosphatidylglycerol (PG), phosphatidylinositols (PI), phosphatidylserines (PS), sulfatides (S), sphingomyelins (SM), triacylglycerides (TG).

	Ion mode (+ve/-ve)	Ions	Retention time (mins)	Internal standard used
Carn_(00:0)	+ve	162.1125	0.30	IS_Car_4:0-d7
Carn_(02:0)	+ve	204.1230	0.30	IS_Car_4:0-d7
Carn_(03:0)	+ve	218.1387	0.30	IS_Car_4:0-d7
Carn_(03:0-2COOH)	+ve	248.1129	0.30	IS_Car_4:0-d7
Carn_(03:1)	+ve	216.1230	0.28	IS_Car_4:0-d7
Carn_(03:0-OH)	+ve	234.1336	0.30	IS_Car_4:0-d7
Carn_(04:0)	+ve	232.1543	0.30	IS_Car_4:0-d7
Carn_(04:0-2COOH)	+ve	262.1285	0.28	IS_Car_4:0-d7
Carn_(04:1)	+ve	230.1387	0.30	IS_Car_4:0-d7
Carn_(04:0-OH)	+ve	248.1493	0.30	IS_Car_4:0-d7
Carn_(05:0)	+ve	246.1700	0.30	IS_Car_4:0-d7
Carn_(05:1)	+ve	244.1543	0.30	IS_Car_4:0-d7
Carn_(06:0)	+ve	260.1856	0.30	IS_Car_4:0-d7
Carn_(06:0-2COOH)	+ve	290.1598	0.30	IS_Car_4:0-d7
Carn_(06:1)	+ve	258.1700	0.30	IS_Car_4:0-d7
Carn_(07:0)	+ve	274.2013	0.30	IS_Car_4:0-d7
Carn_(08:0)	+ve	288.2169	0.30	IS_Car_4:0-d7
Carn_(08:0-2COOH)	+ve	317.1838	0.30	IS_Car_4:0-d7
Carn_(08:1)	+ve	286.2013	0.30	IS_Car_4:0-d7
Carn_(09:0)	+ve	302.2326	0.30	IS_Car_16:0-d3
Carn_(10:0)	+ve	316.2482	0.30	IS_Car_16:0-d3
Carn_(10:0-2COOH)	+ve	346.2224	0.30	IS_Car_16:0-d3
Carn_(10:1)	+ve	314.2326	0.30	IS_Car_16:0-d3
Carn_(10:2)	+ve	298.2377	0.30	IS_Car_16:0-d3
Carn_(10:0-OH)	+ve	332.2432	0.30	IS_Car_16:0-d3
Carn_(11:0)	+ve	330.2639	0.30	IS_Car_16:0-d3
Carn_(12:0)	+ve	344.2795	0.40	IS_Car_16:0-d3
Carn_(12:1)	+ve	342.2639	0.30	IS_Car_16:0-d3
Carn_(12:0-OH)	+ve	360.2745	0.30	IS_Car_16:0-d3
Carn_(13:0)	+ve	358.2952	0.40	IS_Car_16:0-d3
Carn_(14:0)	+ve	372.3108	0.46	IS_Car_16:0-d3
Carn_(14:1)	+ve	370.2952	0.40	IS_Car_16:0-d3
Carn_(14:2)	+ve	354.3003	0.30	IS_Car_16:0-d3
Carn_(14:0-OH)	+ve	388.3058	0.40	IS_Car_16:0-d3
Carn_(15:0)	+ve	386.3265	0.46	IS_Car_16:0-d3
Carn_(16:0)	+ve	400.3421	0.49	IS_Car_16:0-d3
Carn_(16:1)	+ve	389.3265	0.40	IS_Car_16:0-d3
Carn_(16:2)	+ve	396.3108	0.40	IS_Car_16:0-d3
Carn_(16:0-OH)	+ve	416.3371	0.46	IS_Car_16:0-d3
Carn_(17:0)	+ve	414.3578	0.54	IS_Car_16:0-d3
Carn_(18:0)	+ve	428.3734	0.62	IS_Car_16:0-d3
Carn_(18:1)	+ve	426.3578	0.51	IS_Car_16:0-d3
Carn_(18:2)	+ve	424.3421	0.46	IS_Car_16:0-d3
Carn_(18:3)	+ve	422.3265	0.46	IS_Car_16:0-d3

Carn_(18:0-OH)	+ve	444.3684	0.50	IS_Car_16:0-d3
Carn_(19:0)	+ve	442.3891	0.65	IS_Car_16:0-d3
Carn_(20:0)	+ve	456.4047	0.87	IS_Car_16:0-d3
Carn_(22:5)	+ve	474.3578	0.46	IS_Car_16:0-d3
Cer_(32:0)	+ve	494.4931, 512.5037	3.53	IS_Cer_16:0-d31
Cer_(32:0-OH)	+ve	528.4986	3.21	IS_Cer_16:0-d31
Cer_(32:1)	+ve	492.4775, 510.4881	3.25	IS_Cer_16:0-d31
Cer_(32:1-OH)	+ve	526.4830	2.91	IS_Cer_16:0-d31
Cer_(33:0)	+ve	508.5088, 526.5194	3.87	IS_Cer_16:0-d31
Cer_(33:0-OH)	+ve	542.5143	3.61	IS_Cer_16:0-d31
Cer_(33:1)	+ve	506.4931, 524.5037	3.65	IS_Cer_16:0-d31
Cer_(33:1-OH)	+ve	540.5986	3.30	IS_Cer_16:0-d31
Cer_(34:0)	+ve	522.5244, 540.5350	4.13	IS_Cer_16:0-d31
Cer_(34:0-OH)	+ve	556.5299	3.93	IS_Cer_16:0-d31
Cer_(34:1)	+ve	520.5088, 538.5194	3.89	IS_Cer_16:0-d31
Cer_(34:1-OH)	+ve	554.5143	3.71	IS_Cer_16:0-d31
Cer_(35:0)	+ve	536.5401, 554.5507	4.33	IS_Cer_16:0-d31
Cer_(35:0-OH)	+ve	570.5456	4.15	IS_Cer_16:0-d31
Cer_(35:1)	+ve	534.5244, 552.5350	4.19	IS_Cer_16:0-d31
Cer_(35:1-OH)	+ve	568.5299	3.99	IS_Cer_16:0-d31
Cer_(36:0)	+ve	550.5557, 568.5663	4.49	IS_Cer_16:0-d31
Cer_(36:0-OH)	+ve	584.5612	4.36	IS_Cer_16:0-d31
Cer_(36:1)	+ve	548.5401, 566.5507	4.37	IS_Cer_16:0-d31
Cer_(36:1-OH)	+ve	582.5456	4.21	IS_Cer_16:0-d31
Cer_(36:2)	+ve	546.5244, 564.5350	4.05	IS_Cer_16:0-d31
Cer_(36:2-OH)	+ve	580.5299	3.83	IS_Cer_16:0-d31
Cer_(37:0)	+ve	564.5714, 582.5820	4.65	IS_Cer_16:0-d31
Cer_(37:0-OH)	+ve	598.5769	4.52	IS_Cer_16:0-d31
Cer_(37:1)	+ve	562.5557, 580.5663	4.55	IS_Cer_16:0-d31
Cer_(37:1-OH)	+ve	596.5612	4.38	IS_Cer_16:0-d31
Cer_(37:2)	+ve	560.5401, 578.5507	4.25	IS_Cer_16:0-d31
Cer_(38:0)	+ve	578.5870, 596.5976	4.79	IS_Cer_16:0-d31
Cer_(38:0-OH)	+ve	612.5925	4.66	IS_Cer_16:0-d31
Cer_(38:1)	+ve	576.5714, 594.5820	4.69	IS_Cer_16:0-d31
Cer_(38:1-OH)	+ve	610.5769	4.54	IS_Cer_16:0-d31
Cer_(38:2)	+ve	574.5557, 592.5663	4.39	IS_Cer_16:0-d31
Cer_(39:0)	+ve	592.6027, 610.6133	4.90	IS_Cer_16:0-d31
Cer_(39:0-OH)	+ve	626.6082	4.80	IS_Cer_16:0-d31
Cer_(39:1)	+ve	590.5870, 608.5976	4.83	IS_Cer_16:0-d31
Cer_(39:1-OH)	+ve	624.5925	4.70	IS_Cer_16:0-d31
Cer_(39:2)	+ve	588.5714, 606.5820	4.55	IS_Cer_16:0-d31
Cer_(40:0)	+ve	606.6183, 624.6289	5.04	IS_Cer_16:0-d31
Cer_(40:0-OH)	+ve	640.6238	4.94	IS_Cer_16:0-d31
Cer_(40:1)	+ve	604.6027, 622.6133	4.96	IS_Cer_16:0-d31
Cer_(40:1-OH)	+ve	638.6082	4.83	IS_Cer_16:0-d31
Cer_(40:2)	+ve	602.5870, 620.5976	4.69	IS_Cer_16:0-d31
Cer_(41:0)	+ve	620.6340, 638.6446	5.17	IS_Cer_16:0-d31
Cer_(41:0-OH)	+ve	654.6395	5.06	IS_Cer_16:0-d31
Cer_(41:1)	+ve	618.6183, 636.6289	5.08	IS_Cer_16:0-d31
Cer_(41:1-OH)	+ve	652.6238	4.96	IS_Cer_16:0-d31
Cer_(41:2)	+ve	616.6027, 634.6133	4.83	IS_Cer_16:0-d31
Cer_(42:0)	+ve	634.6496, 652.6602	5.29	IS_Cer_16:0-d31
Cer_(42:0-OH)	+ve	668.6551	5.19	IS_Cer_16:0-d31
Cer_(42:1)	+ve	632.6340, 650.6446	5.19	IS_Cer_16:0-d31
Cer_(42:1-OH)	+ve	666.6395	4.92	IS_Cer_16:0-d31
Cer_(42:2)	+ve	630.6183, 648.6289	4.94	IS_Cer_16:0-d31
Cer_(42:3)	+ve	628.6027, 646.6133	4.87	IS_Cer_16:0-d31
Cer_(43:0)	+ve	648.6653, 666.6759	5.41	IS_Cer_16:0-d31

Cer_(43:0-OH)	+ve	682.6708	5.31	IS_Cer_16:0-d31
Cer_(43:1)	+ve	646.6496, 664.6602	5.31	IS_Cer_16:0-d31
Cer_(43:1-OH)	+ve	680.6551	5.21	IS_Cer_16:0-d31
Cer_(43:2)	+ve	644.6340, 662.6446	5.08	IS_Cer_16:0-d31
Cer_(44:0)	+ve	662.6809, 680.6915	5.53	IS_Cer_16:0-d31
Cer_(44:0-OH)	+ve	696.6864	5.43	IS_Cer_16:0-d31
Cer_(44:1)	+ve	660.6653, 678.6759	5.41	IS_Cer_16:0-d31
Cer_(44:1-OH)	+ve	694.6708	5.17	IS_Cer_16:0-d31
Cer_(44:2)	+ve	658.6496, 676.6602	5.19	IS_Cer_16:0-d31
Cer_(45:0)	+ve	676.6966, 694.7072	5.65	IS_Cer_16:0-d31
Cer_(45:0-OH)	+ve	710.7021	5.53	IS_Cer_16:0-d31
Cer_(45:1)	+ve	674.6809, 692.6915	5.39	IS_Cer_16:0-d31
Cer_(45:1-OH)	+ve	708.6864	5.43	IS_Cer_16:0-d31
Cer_(45:2)	+ve	690.6759, 672.6653	5.31	IS_Cer_16:0-d31
Cer_(46:0)	+ve	690.7122, 708.7228	5.75	IS_Cer_16:0-d31
Cer_(46:0-OH)	+ve	724.7177	5.63	IS_Cer_16:0-d31
Cer_(46:1)	+ve	688.6966, 706.7072	5.50	IS_Cer_16:0-d31
Cer_(46:1-OH)	+ve	722.7021	5.43	IS_Cer_16:0-d31
Cer_(46:2)	+ve	686.6809, 704.6915	5.41	IS_Cer_16:0-d31
Cer_(47:0)	+ve	704.7279, 722.7385	5.88	IS_Cer_16:0-d31
Cer_(47:0-OH)	+ve	738.7334	5.65	IS_Cer_16:0-d31
Cer_(47:1)	+ve	702.7122, 720.7228	5.76	IS_Cer_16:0-d31
Cer_(48:0)	+ve	718.7435, 736.7541	5.96	IS_Cer_16:0-d31
Cer_(48:0-OH)	+ve	752.7490	5.76	IS_Cer_16:0-d31
Cer_(48:1)	+ve	716.7279, 734.7385	5.75	IS_Cer_16:0-d31
Cer_(48:1-OH)	+ve	750.7334	5.61	IS_Cer_16:0-d31
Cer_(48:2)	+ve	714.7122, 732.7228	5.53	IS_Cer_16:0-d31
Cer_(50:0)	+ve	746.7748, 764.7854	6.08	IS_Cer_16:0-d31
Cer_(50:0-OH)	+ve	780.7803	5.91	IS_Cer_16:0-d31
Cer_(50:1)	+ve	744.7592, 762.7698	5.90	IS_Cer_16:0-d31
Cer_(50:2)	+ve	742.7435, 760.7541	5.80	IS_Cer_16:0-d31
CL_(66:02)	-ve	1375.9650	5.64	IS_TG_45:0-d87
CL_(66:03)	-ve	1373.9493	5.50	IS_TG_45:0-d87
CL_(66:04)	-ve	1371.9337	5.36	IS_TG_45:0-d87
CL_(66:05)	-ve	1396.9180	5.25	IS_TG_45:0-d87
CL_(66:06)	-ve	1367.9024	5.06	IS_TG_45:0-d87
CL_(66:07)	-ve	1365.8867	4.99	IS_TG_45:0-d87
CL_(67:02)	-ve	1389.9806	5.72	IS_TG_45:0-d87
CL_(67:03)	-ve	1387.9650	5.58	IS_TG_45:0-d87
CL_(67:05)	-ve	1383.9337	5.25	IS_TG_45:0-d87
CL_(68:00)	-ve	1408.0276	5.98	IS_TG_45:0-d87
CL_(68:01)	-ve	1406.0119	5.80	IS_TG_45:0-d87
CL_(68:02)	-ve	1403.9963	5.80	IS_TG_45:0-d87
CL_(68:03)	-ve	1401.9806	5.66	IS_TG_45:0-d87
CL_(68:04)	-ve	1399.9650	5.50	IS_TG_45:0-d87
CL_(69:04)	-ve	1413.9806	5.60	IS_TG_45:0-d87
CL_(69:05)	-ve	1411.9650	5.44	IS_TG_45:0-d87
CL_(69:06)	-ve	1409.9493	5.31	IS_TG_45:0-d87
CL_(69:07)	-ve	1407.9337	5.16	IS_TG_45:0-d87
CL_(70:00)	-ve	1436.0589	6.07	IS_TG_45:0-d87
CL_(70:01)	-ve	1434.0432	5.98	IS_TG_45:0-d87
CL_(70:02)	-ve	1432.0276	5.84	IS_TG_45:0-d87
CL_(70:03)	-ve	1430.0119	5.82	IS_TG_45:0-d87
CL_(70:04)	-ve	1427.9963	5.68	IS_TG_45:0-d87
CL_(70:05)	-ve	1425.9806	5.52	IS_TG_45:0-d87
CL_(70:06)	-ve	1423.9650	5.38	IS_TG_45:0-d87
CL_(70:07)	-ve	1421.9493	5.22	IS_TG_45:0-d87
CL_(70:08)	-ve	1419.9337	5.12	IS_TG_45:0-d87

CL_(70:09)	-ve	1417.9180	5.20	IS_TG_45:0-d87
CL_(71:02)	-ve	1446.0432	5.90	IS_TG_45:0-d87
CL_(71:03)	-ve	1444.0276	5.72	IS_TG_45:0-d87
CL_(71:04)	-ve	1442.0119	5.72	IS_TG_45:0-d87
CL_(71:05)	-ve	1439.9963	5.46	IS_TG_45:0-d87
CL_(71:06)	-ve	1437.9806	5.46	IS_TG_45:0-d87
CL_(71:07)	-ve	1435.9650	5.32	IS_TG_45:0-d87
CL_(71:08)	-ve	1433.9493	5.18	IS_TG_45:0-d87
CL_(72:00)	-ve	1464.0902	6.14	IS_TG_45:0-d87
CL_(72:01)	-ve	1462.0745	6.05	IS_TG_45:0-d87
CL_(72:02)	-ve	1460.0589	6.00	IS_TG_45:0-d87
CL_(72:03)	-ve	1458.0432	5.84	IS_TG_45:0-d87
CL_(72:04)	-ve	1456.0276	5.70	IS_TG_45:0-d87
CL_(72:05)	-ve	1454.0119	5.64	IS_TG_45:0-d87
CL_(72:06)	-ve	1451.9963	5.54	IS_TG_45:0-d87
CL_(72:07)	-ve	1449.9806	5.40	IS_TG_45:0-d87
CL_(72:08)	-ve	1447.9650	5.27	IS_TG_45:0-d87
CL_(72:09)	-ve	1445.9493	5.22	IS_TG_45:0-d87
CL_(72:10)	-ve	1443.9337	5.16	IS_TG_45:0-d87
CL_(74:06)	-ve	1480.0276	5.70	IS_TG_45:0-d87
CL_(74:07)	-ve	1478.0119	5.56	IS_TG_45:0-d87
CL_(74:08)	-ve	1475.9963	5.44	IS_TG_45:0-d87
CL_(74:09)	-ve	1473.9806	5.32	IS_TG_45:0-d87
CL_(74:10)	-ve	1471.9650	5.42	IS_TG_45:0-d87
CL_(74:11)	-ve	1469.9493	5.31	IS_TG_45:0-d87
CL_(76:09)	-ve	1502.0119	5.60	IS_TG_45:0-d87
CL_(76:10)	-ve	1499.9963	5.56	IS_TG_45:0-d87
CL_(76:11)	-ve	1497.9806	5.46	IS_TG_45:0-d87
CL_(76:12)	-ve	1495.9650	5.34	IS_TG_45:0-d87
DG_(32:0)	+ve	551.5034, 591.4959, 607.4698	4.63	IS_TG_45:0-d87
DG_(34:0)	+ve	579.5347, 619.5272, 635.5011	4.89	IS_TG_45:0-d87
DG_(34:1)	+ve	577.5190, 617.5115, 633.4855	4.65	IS_TG_45:0-d87
DG_(36:0)	+ve	647.5585, 663.5324, 607.5660	5.14	IS_TG_45:0-d87
DG_(36:1)	+ve	605.5503, 645.5428, 661.5168	4.92	IS_TG_45:0-d87
DG_(36:2)	+ve	603.5347, 643.5272, 659.5011	4.68	IS_TG_45:0-d87
GM1_(32:0)	-ve	1490.8224	1.23	IS_PG_34:1-d31
GM1_(32:1)	-ve	1488.8068	1.15	IS_PG_34:1-d31
GM1_(34:0)	-ve	1518.8538	1.95	IS_PG_34:1-d31
GM1_(34:1)	-ve	1516.8381	1.89	IS_PG_34:1-d31
GM1_(34:1-OH)	-ve	1532.8330	1.90	IS_PG_34:1-d31
GM1_(35:0)	-ve	1532.8694	2.28	IS_PG_34:1-d31
GM1_(35:1)	-ve	1530.8538	2.22	IS_PG_34:1-d31
GM1_(36:0)	-ve	1546.8850	2.62	IS_PG_34:1-d31
GM1_(36:1)	-ve	1544.8694	2.57	IS_PG_34:1-d31
GM1_(36:1-OH)	-ve	1560.8643	2.42	IS_PG_34:1-d31
GM1_(36:2)	-ve	1542.8537	2.07	IS_PG_34:1-d31
GM1_(37:0)	-ve	1560.9007	2.94	IS_PG_34:1-d31
GM1_(37:1)	-ve	1558.8850	2.89	IS_PG_34:1-d31
GM1_(37:2)	-ve	1556.8694	2.38	IS_PG_34:1-d31
GM1_(38:0)	-ve	1574.9163	3.30	IS_PG_34:1-d31
GM1_(38:1)	-ve	1572.9007	3.24	IS_PG_34:1-d31
GM1_(38:1-OH)	-ve	1588.8956	3.13	IS_PG_34:1-d31
GM1_(38:2)	-ve	1570.8850	2.70	IS_PG_34:1-d31
GM1_(39:0)	-ve	1588.9320	3.69	IS_PG_34:1-d31
GM1_(39:1)	-ve	1586.9163	3.61	IS_PG_34:1-d31
GM1_(39:1-OH)	-ve	1602.9112	3.52	IS_PG_34:1-d31
GM1_(40:0)	-ve	1602.9476	3.93	IS_PG_34:1-d31
GM1_(40:1)	-ve	1600.9320	3.84	IS_PG_34:1-d31

GM1_(40:1-OH)	-ve	1616.9269	3.72	IS_PG_34:1-d31
Hex-Cer_(32:0)	+ve	656.5460, 674.5565	2.85	IS_Cer_16:0-d31
Hex-Cer_(32:1)	+ve	654.5303, 672.5409	2.59	IS_Cer_16:0-d31
Hex-Cer_(34:0)	+ve	684.5773, 702.5878	3.61	IS_Cer_16:0-d31
Hex-Cer_(34:0-OH)	+ve	718.5828	3.41	IS_Cer_16:0-d31
Hex-Cer_(34:1)	+ve	682.5616, 700.5722	3.30	IS_Cer_16:0-d31
Hex-Cer_(34:1-OH)	+ve	716.5671	3.13	IS_Cer_16:0-d31
Hex-Cer_(34:2)	+ve	680.5460, 698.5565	3.13	IS_Cer_16:0-d31
Hex-Cer_(34:2-OH)	+ve	714.5515	2.75	IS_Cer_16:0-d31
Hex-Cer_(35:0)	+ve	698.5929, 716.6035	3.93	IS_Cer_16:0-d31
Hex-Cer_(35:0-OH)	+ve	732.5984	3.79	IS_Cer_16:0-d31
Hex-Cer_(35:1)	+ve	696.5773, 714.5878	3.71	IS_Cer_16:0-d31
Hex-Cer_(35:1-OH)	+ve	730.5828	3.51	IS_Cer_16:0-d31
Hex-Cer_(35:2)	+ve	694.5616, 712.5722	3.51	IS_Cer_16:0-d31
Hex-Cer_(36:0)	+ve	712.6086, 730.6191	4.14	IS_Cer_16:0-d31
Hex-Cer_(36:0-OH)	+ve	746.6141	4.04	IS_Cer_16:0-d31
Hex-Cer_(36:1)	+ve	710.5929, 728.6035	3.98	IS_Cer_16:0-d31
Hex-Cer_(36:1-OH)	+ve	744.5984	3.91	IS_Cer_16:0-d31
Hex-Cer_(36:2)	+ve	708.5773, 726.5878	3.85	IS_Cer_16:0-d31
Hex-Cer_(36:2-OH)	+ve	742.5828	3.43	IS_Cer_16:0-d31
Hex-Cer_(37:0)	+ve	726.6242, 744.6348	4.34	IS_Cer_16:0-d31
Hex-Cer_(37:0-OH)	+ve	760.6297	4.26	IS_Cer_16:0-d31
Hex-Cer_(37:1)	+ve	724.6086, 742.6191	4.20	IS_Cer_16:0-d31
Hex-Cer_(37:1-OH)	+ve	758.6141	4.08	IS_Cer_16:0-d31
Hex-Cer_(37:2)	+ve	722.5929, 740.6035	4.08	IS_Cer_16:0-d31
Hex-Cer_(38:0)	+ve	740.6399, 758.6504	4.51	IS_Cer_16:0-d31
Hex-Cer_(38:0-OH)	+ve	774.6454	4.43	IS_Cer_16:0-d31
Hex-Cer_(38:1)	+ve	738.6242, 756.6348	4.38	IS_Cer_16:0-d31
Hex-Cer_(38:1-OH)	+ve	772.6297	4.30	IS_Cer_16:0-d31
Hex-Cer_(38:2)	+ve	736.6086, 754.6191	4.30	IS_Cer_16:0-d31
Hex-Cer_(39:0)	+ve	754.6555, 772.6661	4.63	IS_Cer_16:0-d31
Hex-Cer_(39:0-OH)	+ve	788.6610	4.58	IS_Cer_16:0-d31
Hex-Cer_(39:1)	+ve	752.6399, 770.6504	4.54	IS_Cer_16:0-d31
Hex-Cer_(39:2)	+ve	750.6242, 768.6348	4.47	IS_Cer_16:0-d31
Hex-Cer_(40:0)	+ve	768.6712, 786.6817	4.77	IS_Cer_16:0-d31
Hex-Cer_(40:0-OH)	+ve	802.6767	4.72	IS_Cer_16:0-d31
Hex-Cer_(40:1)	+ve	766.6555, 784.6661	4.69	IS_Cer_16:0-d31
Hex-Cer_(40:1-OH)	+ve	800.6610	4.62	IS_Cer_16:0-d31
Hex-Cer_(40:2)	+ve	764.6399, 782.6504	4.62	IS_Cer_16:0-d31
Hex-Cer_(40:2-OH)	+ve	798.6454	4.30	IS_Cer_16:0-d31
Hex-Cer_(41:0)	+ve	782.6868, 800.6974	4.90	IS_Cer_16:0-d31
Hex-Cer_(41:0-OH)	+ve	816.6923	4.85	IS_Cer_16:0-d31
Hex-Cer_(41:1)	+ve	780.6712, 798.6817	4.83	IS_Cer_16:0-d31
Hex-Cer_(41:2)	+ve	796.6661, 778.6555	4.76	IS_Cer_16:0-d31
Hex-Cer_(42:0)	+ve	796.7025, 814.7130	5.02	IS_Cer_16:0-d31
Hex-Cer_(42:0-OH)	+ve	830.7080	4.98	IS_Cer_16:0-d31
Hex-Cer_(42:1)	+ve	794.6868, 812.6974	4.94	IS_Cer_16:0-d31
Hex-Cer_(42:1-OH)	+ve	828.6923	4.88	IS_Cer_16:0-d31
Hex-Cer_(42:2)	+ve	792.6712, 810.6817	4.67	IS_Cer_16:0-d31
Hex-Cer_(42:2-OH)	+ve	826.6767	4.60	IS_Cer_16:0-d31
Hex-Cer_(43:0)	+ve	810.7181, 828.7287	5.08	IS_Cer_16:0-d31
Hex-Cer_(43:0-OH)	+ve	844.7236	4.96	IS_Cer_16:0-d31
Hex-Cer_(43:1)	+ve	808.7025, 826.7130	4.88	IS_Cer_16:0-d31
Hex-Cer_(43:2)	+ve	806.6868, 824.6974	4.81	IS_Cer_16:0-d31
Hex-Cer_(44:0)	+ve	824.7338, 842.7443	5.26	IS_Cer_16:0-d31
Hex-Cer_(44:1)	+ve	822.7181, 840.7287	5.02	IS_Cer_16:0-d31
Hex-Cer_(44:2)	+ve	820.7025, 838.7130	4.92	IS_Cer_16:0-d31
LPC_(12:0)	+ve	440.2772	0.40	IS_LPC_14:0-d42

LPC_(14:0)	+ve	468.3085	0.44	IS_LPC_14:0-d42
LPC_(14:1)	+ve	466.2928	0.40	IS_LPC_14:0-d42
LPC_(15:0)	+ve	482.3241	0.46	IS_LPC_14:0-d42
LPC_(15:1)	+ve	480.3085	0.42	IS_LPC_14:0-d42
LPC_(16:0)	+ve	496.3398	0.51	IS_LPC_14:0-d42
LPC_(16:1)	+ve	494.3241	0.44	IS_LPC_14:0-d42
LPC_(17:0)	+ve	510.3554	0.58	IS_LPC_14:0-d42
LPC_(17:1)	+ve	508.3398	0.51	IS_LPC_14:0-d42
LPC_(18:0)	+ve	524.3711	0.67	IS_LPC_14:0-d42
LPC_(18:1)	+ve	522.3554	0.54	IS_LPC_14:0-d42
LPC_(18:2)	+ve	520.3398	0.46	IS_LPC_14:0-d42
LPC_(18:3)	+ve	518.3241	0.44	IS_LPC_14:0-d42
LPC_(18:4)	+ve	516.3085	0.43	IS_LPC_14:0-d42
LPC_(19:0)	+ve	538.3867	0.77	IS_LPC_14:0-d42
LPC_(20:0)	+ve	552.4024	0.95	IS_LPC_14:0-d42
LPC_(20:3)	+ve	546.3554	0.75	IS_LPC_14:0-d42
LPC_(20:4)	+ve	544.3398	0.65	IS_LPC_14:0-d42
LPC_(20:5)	+ve	542.3241	0.44	IS_LPC_14:0-d42
LPC_(21:0)	+ve	566.4180	1.15	IS_LPC_14:0-d42
LPC_(22:4)	+ve	572.3711	0.70	IS_LPC_14:0-d42
LPC_(22:5)	+ve	570.3554	0.67	IS_LPC_14:0-d42
LPC_(22:6)	+ve	568.3398	0.66	IS_LPC_14:0-d42
LPE_(14:0)	+ve	426.2615	0.44	IS_LPC_14:0-d42
LPE_(15:0)	+ve	440.2772	0.46	IS_LPC_14:0-d42
LPE_(16:0)	+ve	454.2928	0.53	IS_LPC_14:0-d42
LPE_(16:1)	+ve	452.2772	0.46	IS_LPC_14:0-d42
LPE_(17:0)	+ve	468.3085	0.61	IS_LPC_14:0-d42
LPE_(17:1)	+ve	466.2928	0.50	IS_LPC_14:0-d42
LPE_(18:0)	+ve	482.3241	0.70	IS_LPC_14:0-d42
LPE_(18:1)	+ve	480.3085	0.55	IS_LPC_14:0-d42
LPE_(18:2)	+ve	478.2928	0.46	IS_LPC_14:0-d42
LPE_(18:3)	+ve	476.2772	0.53	IS_LPC_14:0-d42
LPE_(18:4)	+ve	474.2615	0.46	IS_LPC_14:0-d42
LPE_(19:0)	+ve	496.3398	0.82	IS_LPC_14:0-d42
LPE_(20:0)	+ve	510.3554	1.02	IS_LPC_14:0-d42
LPE_(20:3)	+ve	504.3085	0.75	IS_LPC_14:0-d42
LPE_(20:4)	+ve	502.2928	0.57	IS_LPC_14:0-d42
LPE_(20:5)	+ve	500.2772	0.48	IS_LPC_14:0-d42
LPE_(22:4)	+ve	530.3241	0.72	IS_LPC_14:0-d42
LPE_(22:5)	+ve	528.3085	0.63	IS_LPC_14:0-d42
LPE_(22:6)	+ve	526.2928	0.63	IS_LPC_14:0-d42
LPI_(16:0)	-ve	571.2889	0.41	IS_PI_34:1-d31
LPI_(17:0)	-ve	585.3045	0.45	IS_PI_34:1-d31
LPI_(18:0)	-ve	599.3202	0.50	IS_PI_34:1-d31
LPI_(18:1)	-ve	597.3045	0.43	IS_PI_34:1-d31
LPI_(18:2)	-ve	595.2889	0.39	IS_PI_34:1-d31
LPI_(19:0)	-ve	613.3358	0.55	IS_PI_34:1-d31
LPI_(20:0)	-ve	627.3515	0.62	IS_PI_34:1-d31
LPI_(20:1)	-ve	625.3358	0.50	IS_PI_34:1-d31
LPI_(20:2)	-ve	623.3202	0.41	IS_PI_34:1-d31
LPI_(20:3)	-ve	621.3045	0.41	IS_PI_34:1-d31
LPI_(20:4)	-ve	619.2889	0.39	IS_PI_34:1-d31
LPI_(20:5)	-ve	617.2732	0.35	IS_PI_34:1-d31
LPI_(21:0)	-ve	641.3671	0.74	IS_PI_34:1-d31
LPI_(22:0)	-ve	655.3828	0.90	IS_PI_34:1-d31
LPI_(22:1)	-ve	653.3671	0.63	IS_PI_34:1-d31
LPI_(22:4)	-ve	647.3202	0.41	IS_PI_34:1-d31
LPI_(22:5)	-ve	645.3045	0.39	IS_PI_34:1-d31

LPI_(22:6)	-ve	643.2889	0.37	IS_PI_34:1-d31
LPI_(24:1)	-ve	681.3984	0.88	IS_PI_34:1-d31
LPS_(16:0)	-ve	496.2681	0.47	IS_PS_28:0-d54
LPS_(17:0)	-ve	510.2838	0.48	IS_PS_28:0-d54
LPS_(18:0)	-ve	524.2994	0.52	IS_PS_28:0-d54
LPS_(18:1)	-ve	522.2838	0.47	IS_PS_28:0-d54
LPS_(18:2)	-ve	520.2681	0.45	IS_PS_28:0-d54
LPS_(19:0)	-ve	538.3151	0.55	IS_PS_28:0-d54
LPS_(20:0)	-ve	552.3307	0.64	IS_PS_28:0-d54
LPS_(20:1)	-ve	550.3151	0.52	IS_PS_28:0-d54
LPS_(20:2)	-ve	548.2994	0.47	IS_PS_28:0-d54
LPS_(20:3)	-ve	546.2838	0.45	IS_PS_28:0-d54
LPS_(20:4)	-ve	544.2681	0.43	IS_PS_28:0-d54
LPS_(20:5)	-ve	542.2525	0.41	IS_PS_28:0-d54
LPS_(21:0)	-ve	566.3464	0.78	IS_PS_28:0-d54
LPS_(22:0)	-ve	580.3620	0.94	IS_PS_28:0-d54
LPS_(22:1)	-ve	578.3464	0.66	IS_PS_28:0-d54
LPS_(22:2)	-ve	576.3307	0.55	IS_PS_28:0-d54
LPS_(22:3)	-ve	574.3151	0.50	IS_PS_28:0-d54
LPS_(22:4)	-ve	572.2994	0.57	IS_PS_28:0-d54
LPS_(22:5)	-ve	570.2838	0.43	IS_PS_28:0-d54
LPS_(24:0)	-ve	608.3933	1.44	IS_PS_28:0-d54
Lyso_CL_(52:00)	-ve	1169.7979	5.00	IS_TG_45:0-d87
Lyso_CL_(52:01)	-ve	1167.7822	4.67	IS_TG_45:0-d87
Lyso_CL_(52:02)	-ve	1165.7666	4.66	IS_TG_45:0-d87
Lyso_CL_(52:03)	-ve	1163.7509	4.50	IS_TG_45:0-d87
Lyso_CL_(52:04)	-ve	1161.7353	4.30	IS_TG_45:0-d87
Lyso_CL_(52:05)	-ve	1159.7196	4.00	IS_TG_45:0-d87
Lyso_CL_(52:06)	-ve	1157.7040	3.80	IS_TG_45:0-d87
Lyso_CL_(53:00)	-ve	1183.8135	5.26	IS_TG_45:0-d87
Lyso_CL_(53:01)	-ve	1181.7979	5.15	IS_TG_45:0-d87
Lyso_CL_(53:02)	-ve	1179.7822	4.88	IS_TG_45:0-d87
Lyso_CL_(53:03)	-ve	1177.7666	4.69	IS_TG_45:0-d87
Lyso_CL_(53:04)	-ve	1175.7509	4.49	IS_TG_45:0-d87
Lyso_CL_(53:05)	-ve	1173.7353	4.20	IS_TG_45:0-d87
Lyso_CL_(53:06)	-ve	1171.7196	4.00	IS_TG_45:0-d87
Lyso_CL_(54:00)	-ve	1197.8292	5.50	IS_TG_45:0-d87
Lyso_CL_(54:01)	-ve	1195.8135	5.33	IS_TG_45:0-d87
Lyso_CL_(54:02)	-ve	1193.7979	4.88	IS_TG_45:0-d87
Lyso_CL_(54:03)	-ve	1191.7822	4.69	IS_TG_45:0-d87
Lyso_CL_(54:04)	-ve	1189.7666	4.53	IS_TG_45:0-d87
Lyso_CL_(54:05)	-ve	1187.7509	4.39	IS_TG_45:0-d87
Lyso_CL_(54:06)	-ve	1185.7353	4.18	IS_TG_45:0-d87
Lyso_CL_(56:05)	-ve	1215.7822	4.44	IS_TG_45:0-d87
Lyso_CL_(56:06)	-ve	1213.7666	4.43	IS_TG_45:0-d87
MG_(16:0)	+ve	313.2737, 353.2662, 369.2402	0.76	IS_TG_45:0-d87
PA_(30:0)	-ve	619.4344	2.57	IS_PA_34:1-d31
PA_(30:1)	-ve	617.4188	2.03	IS_PA_34:1-d31
PA_(32:0)	-ve	647.4657	3.25	IS_PA_34:1-d31
PA_(32:1)	-ve	645.4501	2.67	IS_PA_34:1-d31
PA_(32:2)	-ve	643.4344	2.15	IS_PA_34:1-d31
PA_(33:1)	-ve	659.4657	3.01	IS_PA_34:1-d31
PA_(34:0)	-ve	675.4970	3.60	IS_PA_34:1-d31
PA_(34:1)	-ve	673.4814	3.36	IS_PA_34:1-d31
PA_(34:2)	-ve	671.4657	2.80	IS_PA_34:1-d31
PA_(34:3)	-ve	669.4501	2.29	IS_PA_34:1-d31
PA_(35:0)	-ve	689.5127	3.88	IS_PA_34:1-d31
PA_(35:1)	-ve	687.4970	3.76	IS_PA_34:1-d31

PA_(35:2)	-ve	685.4814	3.17	IS_PA_34:1-d31
PA_(36:0)	-ve	703.5283	4.02	IS_PA_34:1-d31
PA_(36:1)	-ve	701.5127	3.84	IS_PA_34:1-d31
PA_(36:2)	-ve	699.4970	3.54	IS_PA_34:1-d31
PA_(36:3)	-ve	697.4814	2.89	IS_PA_34:1-d31
PA_(36:4)	-ve	695.4657	2.70	IS_PA_34:1-d31
PA_(38:1)	-ve	729.5440	4.40	IS_PA_34:1-d31
PA_(38:2)	-ve	727.5283	4.04	IS_PA_34:1-d31
PA_(38:3)	-ve	725.5127	3.80	IS_PA_34:1-d31
PA_(38:4)	-ve	723.4970	3.45	IS_PA_34:1-d31
PA_(38:5)	-ve	721.4814	3.01	IS_PA_34:1-d31
PA_(38:6)	-ve	719.4657	2.57	IS_PA_34:1-d31
PA_(40:5)	-ve	749.5127	3.76	IS_PA_34:1-d31
PA_(40:6)	-ve	747.4970	3.25	IS_PA_34:1-d31
PC_(30:0)	+ve	707.5381	3.10	IS_PC_34:1-d31
PC_(30:1)	+ve	704.5225	2.51	IS_PC_34:1-d31
PC_(31:0)	+ve	720.5538	3.57	IS_PC_34:1-d31
PC_(32:0)	+ve	734.5694	3.87	IS_PC_34:1-d31
PC_(32:1)	+ve	732.5538	3.23	IS_PC_34:1-d31
PC_(32:2)	+ve	730.5381	2.65	IS_PC_34:1-d31
PC_(33:0)	+ve	748.5851	4.08	IS_PC_34:1-d31
PC_(33:1)	+ve	746.5694	3.61	IS_PC_34:1-d31
PC_(33:2)	+ve	744.5538	3.00	IS_PC_34:1-d31
PC_(34:0)	+ve	762.6007	4.28	IS_PC_34:1-d31
PC_(34:1)	+ve	760.5851	3.91	IS_PC_34:1-d31
PC_(34:2)	+ve	758.5694	3.40	IS_PC_34:1-d31
PC_(34:3)	+ve	756.5538	2.88	IS_PC_34:1-d31
PC_(34:4)	+ve	754.5381	2.56	IS_PC_34:1-d31
PC_(35:0)	+ve	776.6164	4.41	IS_PC_34:1-d31
PC_(35:1)	+ve	774.6007	4.14	IS_PC_34:1-d31
PC_(35:2)	+ve	772.5851	3.77	IS_PC_34:1-d31
PC_(36:0)	+ve	790.6320	4.58	IS_PC_34:1-d31
PC_(36:1)	+ve	788.6164	4.32	IS_PC_34:1-d31
PC_(36:2)	+ve	786.6007	4.02	IS_PC_34:1-d31
PC_(36:3)	+ve	784.5851	3.48	IS_PC_34:1-d31
PC_(36:4)	+ve	782.5694	3.29	IS_PC_34:1-d31
PC_(37:0)	+ve	804.6477	4.66	IS_PC_34:1-d31
PC_(37:1)	+ve	802.6320	4.48	IS_PC_34:1-d31
PC_(37:2)	+ve	800.6164	4.18	IS_PC_34:1-d31
PC_(37:3)	+ve	798.6007	3.84	IS_PC_34:1-d31
PC_(37:4)	+ve	796.5851	3.72	IS_PC_34:1-d31
PC_(37:5)	+ve	794.5694	3.00	IS_PC_34:1-d31
PC_(37:6)	+ve	792.5538	2.74	IS_PC_34:1-d31
PC_(38:0)	+ve	818.6633	4.84	IS_PC_34:1-d31
PC_(38:1)	+ve	816.6477	4.62	IS_PC_34:1-d31
PC_(38:2)	+ve	814.6320	4.36	IS_PC_34:1-d31
PC_(38:3)	+ve	812.6164	4.16	IS_PC_34:1-d31
PC_(38:4)	+ve	810.6007	3.95	IS_PC_34:1-d31
PC_(38:5)	+ve	808.5851	3.13	IS_PC_34:1-d31
PC_(38:6)	+ve	806.5694	3.11	IS_PC_34:1-d31
PC_(40:2)	+ve	842.6633	4.68	IS_PC_34:1-d31
PC_(40:3)	+ve	840.6477	4.50	IS_PC_34:1-d31
PC_(40:4)	+ve	838.6320	4.24	IS_PC_34:1-d31
PC_(40:5)	+ve	836.6164	4.12	IS_PC_34:1-d31
PC_(40:6)	+ve	834.6007	4.06	IS_PC_34:1-d31
PC_(40:7)	+ve	832.5851	3.91	IS_PC_34:1-d31
PC_C18(plas)-18:1	+ve	772.6214	4.46	IS_PC_34:1-d31
PE_(30:0)	+ve	664.4912	3.32	IS_PE_34:1-d31

PE_(32:0)	+ve	692.5225	4.00	IS_PE_34:1-d31
PE_(32:1)	+ve	690.5068	3.41	IS_PE_34:1-d31
PE_(34:0)	+ve	720.5538	4.39	IS_PE_34:1-d31
PE_(34:1)	+ve	718.5381	4.06	IS_PE_34:1-d31
PE_(34:2)	+ve	716.5225	3.63	IS_PE_34:1-d31
PE_(35:1)	+ve	732.5538	4.23	IS_PE_34:1-d31
PE_(36:0)	+ve	748.5851	4.69	IS_PE_34:1-d31
PE_(36:1)	+ve	746.5694	4.43	IS_PE_34:1-d31
PE_(36:2)	+ve	744.5538	4.13	IS_PE_34:1-d31
PE_(36:3)	+ve	742.5381	3.70	IS_PE_34:1-d31
PE_(36:4)	+ve	740.5225	3.49	IS_PE_34:1-d31
PE_(38:0)	+ve	776.6164	4.89	IS_PE_34:1-d31
PE_(38:1)	+ve	774.6007	4.71	IS_PE_34:1-d31
PE_(38:2)	+ve	772.5851	4.49	IS_PE_34:1-d31
PE_(38:3)	+ve	770.5694	4.24	IS_PE_34:1-d31
PE_(38:4)	+ve	768.5538	4.08	IS_PE_34:1-d31
PE_(38:5)	+ve	766.5381	3.79	IS_PE_34:1-d31
PE_(38:6)	+ve	764.5225	3.29	IS_PE_34:1-d31
PG_(32:0)	-ve	721.5025	2.99	IS_PG_34:1-d31
PG_(33:0)	-ve	735.5182	3.35	IS_PG_34:1-d31
PG_(34:0)	-ve	749.5338	3.71	IS_PG_34:1-d31
PG_(34:1)	-ve	747.5182	3.08	IS_PG_34:1-d31
PG_(35:0)	-ve	763.5495	3.95	IS_PG_34:1-d31
PG_(35:1)	-ve	761.5338	3.45	IS_PG_34:1-d31
PG_(36:0)	-ve	777.5651	4.15	IS_PG_34:1-d31
PG_(36:1)	-ve	775.5495	3.78	IS_PG_34:1-d31
PG_(36:2)	-ve	773.5338	3.26	IS_PG_34:1-d31
PG_(36:3)	-ve	771.5182	2.70	IS_PG_34:1-d31
PG_(36:4)	-ve	769.5025	2.52	IS_PG_34:1-d31
PG_(38:0)	-ve	805.5964	4.47	IS_PG_34:1-d31
PG_(38:1)	-ve	803.5808	4.17	IS_PG_34:1-d31
PG_(38:2)	-ve	801.5651	3.76	IS_PG_34:1-d31
PG_(38:3)	-ve	799.5495	3.49	IS_PG_34:1-d31
PG_(38:4)	-ve	797.5338	3.17	IS_PG_34:1-d31
PG_(38:5)	-ve	795.5182	2.79	IS_PG_34:1-d31
PG_(38:6)	-ve	793.5025	2.39	IS_PG_34:1-d31
PG_(38:7)	-ve	791.4869	2.23	IS_PG_34:1-d31
PG_(40:0)	-ve	833.6277	4.71	IS_PG_34:1-d31
PG_(40:1)	-ve	831.6121	4.49	IS_PG_34:1-d31
PG_(40:2)	-ve	829.5964	4.25	IS_PG_34:1-d31
PG_(40:3)	-ve	827.5808	3.97	IS_PG_34:1-d31
PG_(40:4)	-ve	825.5651	3.67	IS_PG_34:1-d31
PG_(40:5)	-ve	823.5495	3.45	IS_PG_34:1-d31
PG_(40:6)	-ve	821.5338	3.01	IS_PG_34:1-d31
PG_(40:7)	-ve	819.5182	2.69	IS_PG_34:1-d31
PG_(40:8)	-ve	817.5025	2.38	IS_PG_34:1-d31
PG_(42:5)	-ve	851.5808	3.85	IS_PG_34:1-d31
PG_(42:6)	-ve	849.5651	3.45	IS_PG_34:1-d31
PG_(42:7)	-ve	847.5495	3.01	IS_PG_34:1-d31
PG_(42:8)	-ve	845.5338	2.54	IS_PG_34:1-d31
PG_(42:10)	-ve	841.5025	2.80	IS_PG_34:1-d31
PG_(42:11)	-ve	839.4869	2.51	IS_PG_34:1-d31
PI_(32:0)	-ve	809.5186	2.82	IS_PI_34:1-d31
PI_(33:0)	-ve	823.5342	3.18	IS_PI_34:1-d31
PI_(34:0)	-ve	837.5499	3.54	IS_PI_34:1-d31
PI_(34:1)	-ve	835.5342	2.91	IS_PI_34:1-d31
PI_(34:2)	-ve	833.5186	2.42	IS_PI_34:1-d31
PI_(35:0)	-ve	851.5655	3.78	IS_PI_34:1-d31

PI_(35:1)	-ve	849.5499	3.26	IS_PI_34:1-d31
PI_(35:2)	-ve	847.5342	2.76	IS_PI_34:1-d31
PI_(36:0)	-ve	865.5812	4.06	IS_PI_34:1-d31
PI_(36:1)	-ve	863.5655	3.65	IS_PI_34:1-d31
PI_(36:2)	-ve	861.5499	3.11	IS_PI_34:1-d31
PI_(36:3)	-ve	859.5342	2.60	IS_PI_34:1-d31
PI_(36:4)	-ve	857.5186	2.35	IS_PI_34:1-d31
PI_(38:3)	-ve	887.5655	3.31	IS_PI_34:1-d31
PI_(38:4)	-ve	885.5499	3.00	IS_PI_34:1-d31
PI_(38:5)	-ve	883.5342	2.54	IS_PI_34:1-d31
PI_(40:3)	-ve	915.5968	3.88	IS_PI_34:1-d31
PI_(40:4)	-ve	913.5812	3.52	IS_PI_34:1-d31
PI_(40:5)	-ve	911.5655	3.20	IS_PI_34:1-d31
PI_(40:6)	-ve	909.5499	2.86	IS_PI_34:1-d31
PI_(40:8)	-ve	905.5186	1.81	IS_PI_34:1-d31
PS_(32:0)	-ve	734.4978	2.89	IS_PS_28:0-d54
PS_(32:1)	-ve	732.4821	2.32	IS_PS_28:0-d54
PS_(33:0)	-ve	706.4665	3.07	IS_PS_28:0-d54
PS_(33:1)	-ve	746.4978	2.65	IS_PS_28:0-d54
PS_(34:0)	-ve	762.5291	3.38	IS_PS_28:0-d54
PS_(34:1)	-ve	760.5134	2.99	IS_PS_28:0-d54
PS_(34:2)	-ve	758.4978	2.47	IS_PS_28:0-d54
PS_(34:3)	-ve	756.4821	2.02	IS_PS_28:0-d54
PS_(35:0)	-ve	776.5447	3.67	IS_PS_28:0-d54
PS_(35:1)	-ve	774.5291	3.36	IS_PS_28:0-d54
PS_(35:2)	-ve	772.5134	2.81	IS_PS_28:0-d54
PS_(36:0)	-ve	790.5604	4.13	IS_PS_28:0-d54
PS_(36:1)	-ve	788.5447	3.74	IS_PS_28:0-d54
PS_(36:2)	-ve	786.5291	3.16	IS_PS_28:0-d54
PS_(36:3)	-ve	784.5134	2.56	IS_PS_28:0-d54
PS_(38:0)	-ve	818.5917	4.47	IS_PS_28:0-d54
PS_(38:1)	-ve	816.5760	4.17	IS_PS_28:0-d54
PS_(38:2)	-ve	814.5604	3.88	IS_PS_28:0-d54
PS_(38:3)	-ve	812.5447	3.22	IS_PS_28:0-d54
PS_(38:4)	-ve	810.5291	3.18	IS_PS_28:0-d54
PS_(38:5)	-ve	808.5134	3.16	IS_PS_28:0-d54
PS_(38:6)	-ve	806.4978	2.59	IS_PS_28:0-d54
PS_(40:0)	-ve	846.6230	4.73	IS_PS_28:0-d54
PS_(40:1)	-ve	844.6073	4.51	IS_PS_28:0-d54
PS_(40:2)	-ve	842.5917	4.27	IS_PS_28:0-d54
PS_(40:3)	-ve	840.5760	3.71	IS_PS_28:0-d54
PS_(40:4)	-ve	838.5604	3.45	IS_PS_28:0-d54
PS_(40:5)	-ve	836.5447	3.27	IS_PS_28:0-d54
PS_(40:6)	-ve	834.5291	2.94	IS_PS_28:0-d54
PS_(40:7)	-ve	832.5134	2.69	IS_PS_28:0-d54
PS_(42:1)	-ve	872.6386	4.55	IS_PS_28:0-d54
PS_(42:2)	-ve	870.6230	4.28	IS_PS_28:0-d54
PS_(42:3)	-ve	868.6073	4.18	IS_PS_28:0-d54
PS_(42:5)	-ve	864.5760	3.74	IS_PS_28:0-d54
PS_(42:6)	-ve	862.5604	3.22	IS_PS_28:0-d54
PS_(42:9)	-ve	856.5134	2.89	IS_PS_28:0-d54
S_(30:1)	-ve	722.4519	1.01	IS_PG_34:1-d31
S_(32:0)	-ve	752.4988	1.71	IS_PG_34:1-d31
S_(32:1)	-ve	750.4832	1.45	IS_PG_34:1-d31
S_(32:1-OH)	-ve	766.4781	1.31	IS_PG_34:1-d31
S_(32:2)	-ve	748.4675	1.06	IS_PG_34:1-d31
S_(33:0)	-ve	766.5145	2.05	IS_PG_34:1-d31
S_(33:1)	-ve	764.4988	1.80	IS_PG_34:1-d31

S_(33:1-OH)	-ve	780.4937	1.67	IS_PG_34:1-d31
S_(33:2)	-ve	762.4832	1.29	IS_PG_34:1-d31
S_(34:0)	-ve	780.5301	2.39	IS_PG_34:1-d31
S_(34:1)	-ve	778.5145	2.15	IS_PG_34:1-d31
S_(34:1-OH)	-ve	794.5094	2.03	IS_PG_34:1-d31
S_(34:2)	-ve	776.4988	1.59	IS_PG_34:1-d31
S_(35:0)	-ve	794.5458	2.70	IS_PG_34:1-d31
S_(35:1)	-ve	792.5301	2.49	IS_PG_34:1-d31
S_(35:1-OH)	-ve	808.5250	2.35	IS_PG_34:1-d31
S_(35:2)	-ve	790.5145	1.97	IS_PG_34:1-d31
S_(36:0)	-ve	808.5614	3.02	IS_PG_34:1-d31
S_(36:1)	-ve	806.5458	2.79	IS_PG_34:1-d31
S_(36:1-OH)	-ve	822.5407	2.66	IS_PG_34:1-d31
S_(36:2)	-ve	804.5301	2.43	IS_PG_34:1-d31
S_(36:2-OH)	-ve	820.5250	2.15	IS_PG_34:1-d31
S_(37:0)	-ve	822.5771	3.40	IS_PG_34:1-d31
S_(37:1)	-ve	820.5614	3.14	IS_PG_34:1-d31
S_(37:1-OH)	-ve	836.5563	3.01	IS_PG_34:1-d31
S_(37:2)	-ve	818.5458	2.55	IS_PG_34:1-d31
S_(37:2-OH)	-ve	834.5407	2.47	IS_PG_34:1-d31
S_(38:0)	-ve	836.5927	3.75	IS_PG_34:1-d31
S_(38:1)	-ve	834.5771	3.52	IS_PG_34:1-d31
S_(38:1-OH)	-ve	850.5720	3.36	IS_PG_34:1-d31
S_(38:2)	-ve	832.5614	2.85	IS_PG_34:1-d31
S_(38:2-OH)	-ve	848.5563	2.74	IS_PG_34:1-d31
S_(39:0)	-ve	850.6084	3.96	IS_PG_34:1-d31
S_(39:1)	-ve	848.5927	3.83	IS_PG_34:1-d31
S_(39:1-OH)	-ve	864.5876	3.69	IS_PG_34:1-d31
S_(39:2)	-ve	846.5771	3.16	IS_PG_34:1-d31
S_(39:2-OH)	-ve	862.5720	3.02	IS_PG_34:1-d31
S_(40:0)	-ve	864.6240	4.15	IS_PG_34:1-d31
S_(40:1)	-ve	862.6084	4.03	IS_PG_34:1-d31
S_(40:1-OH)	-ve	878.6033	3.96	IS_PG_34:1-d31
S_(40:2)	-ve	860.5927	3.52	IS_PG_34:1-d31
S_(40:2-OH)	-ve	876.5876	3.38	IS_PG_34:1-d31
S_(41:0)	-ve	878.6397	4.29	IS_PG_34:1-d31
S_(41:1)	-ve	876.6240	4.20	IS_PG_34:1-d31
S_(41:2)	-ve	874.6084	3.83	IS_PG_34:1-d31
S_(42:0)	-ve	892.6553	4.43	IS_PG_34:1-d31
S_(42:1)	-ve	890.6397	4.35	IS_PG_34:1-d31
S_(42:1-OH)	-ve	906.6346	4.29	IS_PG_34:1-d31
S_(42:2)	-ve	888.6240	4.01	IS_PG_34:1-d31
S_(42:2-OH)	-ve	904.6186	3.94	IS_PG_34:1-d31
S_(43:0)	-ve	906.6710	4.57	IS_PG_34:1-d31
S_(43:1)	-ve	904.6553	4.49	IS_PG_34:1-d31
S_(43:2)	-ve	902.6397	4.19	IS_PG_34:1-d31
S_(44:0)	-ve	920.6866	4.68	IS_PG_34:1-d31
S_(44:1)	-ve	918.6710	4.60	IS_PG_34:1-d31
S_(44:1-OH)	-ve	934.6659	4.57	IS_PG_34:1-d31
S_(44:2)	-ve	916.6553	4.35	IS_PG_34:1-d31
S_(44:2-OH)	-ve	932.6502	4.28	IS_PG_34:1-d31
S_(45:0)	-ve	934.7023	4.71	IS_PG_34:1-d31
S_(45:1)	-ve	932.6866	4.70	IS_PG_34:1-d31
S_(45:2)	-ve	930.6710	4.47	IS_PG_34:1-d31
S_(46:0)	-ve	948.7179	4.88	IS_PG_34:1-d31
S_(46:1)	-ve	946.7023	4.80	IS_PG_34:1-d31
S_(46:1-OH)	-ve	962.6972	4.78	IS_PG_34:1-d31
S_(46:2)	-ve	944.6866	4.58	IS_PG_34:1-d31

S_(46:2-OH)	-ve	960.6815	4.58	IS_PG_34:1-d31
S_(47:2)	-ve	958.7023	4.70	IS_PG_34:1-d31
S_(48:0)	-ve	976.7492	4.93	IS_PG_34:1-d31
S_(48:1)	-ve	974.7336	4.84	IS_PG_34:1-d31
S_(48:1-OH)	-ve	990.7285	4.82	IS_PG_34:1-d31
S_(48:2)	-ve	972.7179	4.80	IS_PG_34:1-d31
S_(48:2-OH)	-ve	988.7128	4.76	IS_PG_34:1-d31
SM_(30:0)	+ve	649.5279, 671.5098, 687.4838	1.86	IS_SM_34:1-d31
SM_(30:1)	+ve	647.5122, 669.4942, 685.4681	1.60	IS_SM_34:1-d31
SM_(32:0)	+ve	677.5592, 699.5411, 715.5151	2.57	IS_SM_34:1-d31
SM_(32:1)	+ve	675.5435, 697.5255, 713.4994	2.33	IS_SM_34:1-d31
SM_(33:0)	+ve	691.5748, 713.5568, 729.5307	2.93	IS_SM_34:1-d31
SM_(33:1)	+ve	689.5592, 711.5411, 727.5151	2.67	IS_SM_34:1-d31
SM_(34:0)	+ve	705.5905, 727.5725, 743.5464	3.32	IS_SM_34:1-d31
SM_(34:0-OH)	+ve	721.5854	2.91	IS_SM_34:1-d31
SM_(34:1)	+ve	703.5748, 725.5568, 741.5307	3.04	IS_SM_34:1-d31
SM_(34:1-OH)	+ve	719.5698	2.62	IS_SM_34:1-d31
SM_(34:2)	+ve	701.5592, 723.5411, 739.5151	2.47	IS_SM_34:1-d31
SM_(34:2-OH)	+ve	717.5541	2.20	IS_SM_34:1-d31
SM_(35:0)	+ve	719.6061, 741.5881, 757.5620	3.76	IS_SM_34:1-d31
SM_(35:1)	+ve	717.5905, 739.5724, 755.5464	3.44	IS_SM_34:1-d31
SM_(35:2)	+ve	715.5748, 737.5568, 753.5307	2.83	IS_SM_34:1-d31
SM_(36:0)	+ve	733.6218, 755.6037, 771.5777	4.00	IS_SM_34:1-d31
SM_(36:0-OH)	+ve	749.6167	3.84	IS_SM_34:1-d31
SM_(36:1)	+ve	731.6061, 753.5881, 769.5620	3.82	IS_SM_34:1-d31
SM_(36:1-OH)	+ve	747.6011	3.58	IS_SM_34:1-d31
SM_(36:2)	+ve	729.5905, 751.5724, 767.5464	3.19	IS_SM_34:1-d31
SM_(36:3)	+ve	727.5748, 749.5568, 765.5307	2.63	IS_SM_34:1-d31
SM_(37:0)	+ve	747.6374, 769.6194, 785.5933	4.22	IS_SM_34:1-d31
SM_(37:1)	+ve	745.6218, 767.6037, 783.5777	4.06	IS_SM_34:1-d31
SM_(37:2)	+ve	743.6061, 765.5881, 781.5620	3.62	IS_SM_34:1-d31
SM_(38:0)	+ve	761.6531, 783.6350, 799.6090	4.39	IS_SM_34:1-d31
SM_(38:0-OH)	+ve	777.6480	4.34	IS_SM_34:1-d31
SM_(38:1)	+ve	759.6374, 781.6194, 797.5933	4.26	IS_SM_34:1-d31
SM_(38:1-OH)	+ve	775.6324	4.14	IS_SM_34:1-d31
SM_(38:2)	+ve	757.6218, 779.6037, 795.5777	3.94	IS_SM_34:1-d31
SM_(38:3)	+ve	755.6061, 777.5881, 793.5620	3.79	IS_SM_34:1-d31
SM_(39:0)	+ve	775.6687, 797.6507, 813.6246	4.56	IS_SM_34:1-d31
SM_(39:1)	+ve	773.6531, 795.6350, 811.6090	4.45	IS_SM_34:1-d31
SM_(40:0)	+ve	789.6844, 811.6663, 827.6403	4.70	IS_SM_34:1-d31
SM_(40:0-OH)	+ve	805.6793	4.45	IS_SM_34:1-d31
SM_(40:1)	+ve	787.6687, 809.6507, 825.6246	4.39	IS_SM_34:1-d31
SM_(40:1-OH)	+ve	803.6637	4.11	IS_SM_34:1-d31
SM_(40:2)	+ve	785.6531, 807.6350, 823.6090	4.27	IS_SM_34:1-d31
SM_(41:0)	+ve	803.7000, 825.6820, 841.6559	4.82	IS_SM_34:1-d31
SM_(41:1)	+ve	801.6844, 823.6663, 839.6403	4.74	IS_SM_34:1-d31
SM_(42:0)	+ve	817.7157, 839.6976, 855.6716	4.94	IS_SM_34:1-d31
SM_(42:0-OH)	+ve	833.7106	4.74	IS_SM_34:1-d31
SM_(42:1)	+ve	815.7000, 837.6820, 853.6559	4.70	IS_SM_34:1-d31
SM_(42:2)	+ve	813.6844, 835.6663, 851.6403	4.58	IS_SM_34:1-d31
SM_(43:0)	+ve	831.7313, 853.7133, 869.6872	5.04	IS_SM_34:1-d31
SM_(43:1)	+ve	829.7157, 851.6976, 867.6716	4.96	IS_SM_34:1-d31
SM_(44:0)	+ve	845.7470, 867.7289, 883.7029	5.18	IS_SM_34:1-d31
SM_(44:1)	+ve	843.7313, 865.7133, 881.6872	5.10	IS_SM_34:1-d31
SM_(44:2)	+ve	841.7157, 863.6976, 879.6716	4.91	IS_SM_34:1-d31
SM_(45:0)	+ve	859.7626, 881.7446, 897.7185	5.28	IS_SM_34:1-d31
SM_(45:1)	+ve	857.7470, 879.7289, 895.7029	5.18	IS_SM_34:1-d31
SM_(46:0)	+ve	873.7783, 895.7602, 911.7342	5.60	IS_SM_34:1-d31

SM_(46:1)	+ve	871.7626, 893.7446, 909.7185	5.33	IS_SM_34:1-d31
SM_(48:0)	+ve	901.8096, 923.7915, 939.7655	6.00	IS_SM_34:1-d31
SM_(48:2)	+ve	897.7783, 919.7602, 935.7342	5.31	IS_SM_34:1-d31
TG_(06:0)	+ve	219.0863, 236.1129, 241.0683, 257.0422	0.34	IS_TG_45:0-d87
TG_(12:0)	+ve	303.1802, 320.2068, 325.1622, 341.1361	0.42	IS_TG_45:0-d87
TG_(18:0)	+ve	387.2741, 404.3007, 409.2561, 425.2300	0.66	IS_TG_45:0-d87
TG_(24:0)	+ve	471.3680, 488.3946, 493.3500, 509.3239	1.99	IS_TG_45:0-d87
TG_(30:0)	+ve	555.4619, 572.4885, 577.4439, 593.4178	4.01	IS_TG_45:0-d87
TG_(36:0)	+ve	639.5558, 656.5824, 661.5378, 677.5117	5.01	IS_TG_45:0-d87
TG_(44:1)	+ve	749.6654, 766.6919, 771.6473, 787.6212	5.66	IS_TG_45:0-d87
TG_(45:0)	+ve	765.6967, 782.7232, 787.6786, 803.6525	5.95	IS_TG_45:0-d87
TG_(45:1)	+ve	780.7076, 785.6630, 793.6810, 801.6369	5.78	IS_TG_45:0-d87
TG_(45:2)	+ve	761.6654, 778.6919, 783.6473, 799.6212	5.59	IS_TG_45:0-d87
TG_(46:1)	+ve	777.6967, 794.7232, 799.6786, 815.6525	5.89	IS_TG_45:0-d87
TG_(46:2)	+ve	775.6810, 792.7076, 797.6630, 813.6369	5.66	IS_TG_45:0-d87
TG_(46:4)	+ve	771.6497, 788.6763, 793.6317, 809.6056	5.38	IS_TG_45:0-d87
TG_(47:0)	+ve	793.7280, 810.7545, 815.7099, 931.6838	6.16	IS_TG_45:0-d87
TG_(47:1)	+ve	791.7123, 808.7389, 813.6943, 829.6682	5.99	IS_TG_45:0-d87
TG_(47:2)	+ve	789.6967, 806.7232, 811.6786, 827.6525	5.80	IS_TG_45:0-d87
TG_(48:0)	+ve	807.7436, 824.7702, 829.7256, 845.6995	6.30	IS_TG_45:0-d87
TG_(48:1)	+ve	805.7280, 822.7545, 827.7099, 843.6838	6.10	IS_TG_45:0-d87
TG_(48:2)	+ve	803.7123, 820.7340, 825.6900, 841.6682	5.89	IS_TG_45:0-d87
TG_(48:3)	+ve	801.6967, 818.7232, 823.6786, 839.6225	5.72	IS_TG_45:0-d87
TG_(49:0)	+ve	821.7593, 838.7858, 843.7412, 859.7151	6.36	IS_TG_45:0-d87
TG_(49:1)	+ve	819.7436, 836.7702, 841.7256, 857.6995	6.18	IS_TG_45:0-d87
TG_(49:2)	+ve	817.7280, 834.7545, 839.7099, 855.6838	6.01	IS_TG_45:0-d87
TG_(49:3)	+ve	815.7123, 832.7389, 837.6943, 853.6682	5.83	IS_TG_45:0-d87
TG_(50:0)	+ve	835.7749, 852.8015, 857.7569, 873.7308	6.49	IS_TG_45:0-d87
TG_(50:1)	+ve	833.7593, 850.7858, 855.7412, 871.7151	6.30	IS_TG_45:0-d87
TG_(50:2)	+ve	831.7436, 848.7702, 853.7256, 869.6995	6.10	IS_TG_45:0-d87
TG_(50:3)	+ve	829.7280, 846.7545, 851.7099, 867.6838	5.93	IS_TG_45:0-d87
TG_(51:0)	+ve	849.7906, 866.8171, 871.7725, 887.7464	6.59	IS_TG_45:0-d87
TG_(51:1)	+ve	847.7749, 864.8015, 869.7569, 885.7308	6.39	IS_TG_45:0-d87
TG_(51:2)	+ve	845.7593, 862.7858, 867.7412, 883.7151	6.20	IS_TG_45:0-d87
TG_(51:3)	+ve	843.7436, 860.7702, 865.7256, 881.6995	6.03	IS_TG_45:0-d87
TG_(51:4)	+ve	841.7280, 858.7545, 863.7099, 879.6838	5.89	IS_TG_45:0-d87
TG_(52:0)	+ve	863.8062, 880.8328, 885.7882, 901.7621	6.69	IS_TG_45:0-d87
TG_(52:1)	+ve	861.7906, 878.8171, 883.7725, 899.7464	6.50	IS_TG_45:0-d87
TG_(52:2)	+ve	859.7749, 876.8015, 881.7569, 897.7308	6.30	IS_TG_45:0-d87
TG_(52:3)	+ve	857.7593, 874.7858, 879.7412, 895.7151	6.12	IS_TG_45:0-d87
TG_(52:4)	+ve	855.7436, 872.7702, 877.7256, 893.6995	5.95	IS_TG_45:0-d87
TG_(53:0)	+ve	877.8219, 894.8484, 899.8038, 915.7777	6.77	IS_TG_45:0-d87
TG_(53:1)	+ve	875.8062, 892.8328, 897.7882, 913.7621	6.57	IS_TG_45:0-d87
TG_(53:2)	+ve	873.7906, 890.8171, 895.7725, 911.7464	6.39	IS_TG_45:0-d87
TG_(53:3)	+ve	871.7749, 888.8015, 893.7569, 909.7308	6.22	IS_TG_45:0-d87
TG_(53:4)	+ve	869.7593, 886.7858, 891.7412, 907.7151	6.05	IS_TG_45:0-d87
TG_(54:0)	+ve	891.8375, 908.8641, 913.8195, 929.7934	6.86	IS_TG_45:0-d87
TG_(54:1)	+ve	889.8219, 906.8484, 911.8038, 927.7777	6.69	IS_TG_45:0-d87
TG_(54:2)	+ve	887.8062, 904.8328, 909.7882, 925.7621	6.50	IS_TG_45:0-d87
TG_(54:3)	+ve	885.7906, 902.8171, 907.7725, 923.7464	6.32	IS_TG_45:0-d87
TG_(54:4)	+ve	883.7749, 900.8015, 905.7569, 921.7308	6.14	IS_TG_45:0-d87
TG_(54:5)	+ve	881.7593, 898.7858, 903.7412, 919.7151	6.05	IS_TG_45:0-d87
TG_(54:6)	+ve	879.7436, 896.7702, 901.7256, 917.6995	5.91	IS_TG_45:0-d87
TG_(55:0)	+ve	905.8532, 922.8797, 927.8351, 943.8090	6.94	IS_TG_45:0-d87
TG_(55:1)	+ve	903.8375, 920.8641, 925.8195, 941.7934	6.77	IS_TG_45:0-d87
TG_(55:2)	+ve	901.8219, 918.8484, 923.8038, 939.7777	6.59	IS_TG_45:0-d87
TG_(55:3)	+ve	899.8062, 916.8328, 921.7882, 937.7621	6.41	IS_TG_45:0-d87
TG_(55:4)	+ve	897.7906, 914.8171, 919.7725, 935.7464	6.22	IS_TG_45:0-d87

TG_(55:5)	+ve	895.7749, 912.8015, 917.7569, 933.7308	6.14	IS_TG_45:0-d87
TG_(55:6)	+ve	893.7593, 910.7858, 915.7412, 931.7151	6.03	IS_TG_45:0-d87
TG_(56:0)	+ve	919.8688, 936.8954, 941.8508, 957.8247	7.03	IS_TG_45:0-d87
TG_(56:1)	+ve	917.8532, 934.8797, 939.8351, 955.8090	6.86	IS_TG_45:0-d87
TG_(56:2)	+ve	915.8375, 932.8641, 937.8195, 953.7934	6.68	IS_TG_45:0-d87
TG_(56:3)	+ve	913.8219, 930.8484, 935.8038, 951.7777	6.50	IS_TG_45:0-d87
TG_(56:6)	+ve	907.7749, 924.8015, 929.7569, 945.7308	6.07	IS_TG_45:0-d87
TG_(56:7)	+ve	905.7593, 922.7858, 927.7412, 943.7151	5.97	IS_TG_45:0-d87
TG_(56:8)	+ve	903.7436, 920.7702, 925.7256, 941.6995	5.78	IS_TG_45:0-d87
TG_(57:0)	+ve	933.8845, 950.9110, 955.8664, 971.8403	7.09	IS_TG_45:0-d87
TG_(57:2)	+ve	929.8532, 946.8797, 951.8351, 967.8090	6.78	IS_TG_45:0-d87
TG_(57:3)	+ve	927.8375, 944.8641, 949.8195, 965.7934	6.61	IS_TG_45:0-d87
TG_(57:4)	+ve	925.8219, 942.8484, 947.8038, 963.7777	6.42	IS_TG_45:0-d87
TG_(57:5)	+ve	923.8062, 940.8328, 945.7882, 961.7621	6.26	IS_TG_45:0-d87
TG_(57:6)	+ve	921.7906, 938.8171, 943.7725, 959.7464	6.16	IS_TG_45:0-d87
TG_(58:7)	+ve	933.7906, 950.8171, 955.7725, 971.7464	6.18	IS_TG_45:0-d87
TG_(58:8)	+ve	931.7749, 948.8015, 953.7569, 969.7308	5.99	IS_TG_45:0-d87
TG_(58:9)	+ve	929.7593, 946.7858, 951.7412, 967.7151	5.82	IS_TG_45:0-d87
TG_(58:10)	+ve	927.7436, 944.7702, 949.7256, 965.6995	5.66	IS_TG_45:0-d87
TG_(58:12)	+ve	923.7123, 940.7389, 945.6943, 961.6682	5.40	IS_TG_45:0-d87
TG_(59:3)	+ve	955.8688, 972.8954, 977.8508, 993.8247	6.78	IS_TG_45:0-d87
TG_(59:4)	+ve	953.8532, 970.8797, 975.8351, 991.8090	6.61	IS_TG_45:0-d87
TG_(59:5)	+ve	951.8375, 968.8641, 973.8195, 989.7934	6.43	IS_TG_45:0-d87
TG_(59:6)	+ve	949.8219, 966.8484, 971.8038, 987.7777	6.36	IS_TG_45:0-d87
TG_(59:7)	+ve	947.8062, 964.8328, 969.7882, 985.7621	6.26	IS_TG_45:0-d87
TG_(59:8)	+ve	945.7906, 962.8171, 967.7725, 983.7464	6.10	IS_TG_45:0-d87
TG_(59:9)	+ve	943.7749, 960.8015, 965.7569, 981.7308	5.93	IS_TG_45:0-d87
TG_(60:10)	+ve	955.7749, 972.8015, 977.7569, 993.7308	5.83	IS_TG_45:0-d87
TG_(60:12)	+ve	951.7436, 968.7702, 973.7256, 989.6995	5.63	IS_TG_45:0-d87
TG_(61:12)	+ve	965.7593, 982.7858, 987.7412, 1003.7151	5.66	IS_TG_45:0-d87
TG_(62:12)	+ve	979.7749, 996.8015, 1001.7569, 1017.7308	5.78	IS_TG_45:0-d87
TG_(62:13)	+ve	977.7593, 994.7858, 999.7412, 1015.7151	5.64	IS_TG_45:0-d87
TG_(62:14)	+ve	975.7436, 992.7702, 997.7256, 1013.6995	5.43	IS_TG_45:0-d87
TG_(66:18)	+ve	1023.7436, 1040.7702, 1045.7256, 1061.6995	5.34	IS_TG_45:0-d87

© 2020 by the authors. Submitted for possible open access publication under the terms and conditions of the Creative Commons Attribution (CC BY) license (http://creativecommons.org/licenses/by/4.0/).
